# Direct observation of multiple conformational states in Cytochrome P450 oxidoreductase and their modulation by membrane environment and ionic strength

**DOI:** 10.1038/s41598-018-24922-x

**Published:** 2018-05-01

**Authors:** Krutika Bavishi, Darui Li, Stine Eiersholt, Emma N. Hooley, Troels C. Petersen, Birger Lindberg Møller, Nikos S. Hatzakis, Tomas Laursen

**Affiliations:** 1 Plant Biochemistry Laboratory, Department of Plant and Environmental Sciences, University of Copenhagen, Thorvaldsensvej 40, Frederiksberg C, 1871 Denmark; 2 VILLUM Center for Plant Plasticity, Department of Plant and Environmental Sciences, University of Copenhagen, Thorvaldsensvej 40, Frederiksberg C, 1871 Denmark; 3 bioSYNergy, Center for Synthetic Biology, University of Copenhagen, Thorvaldsensvej 40, Frederiksberg C, 1871 Denmark; 4 Department of Chemistry & Nanoscience Center, Thorvaldsensvej 40, University of Copenhagen, Frederiksberg C, 1871 Denmark; 5 The Nano Spectroscopy Group, Nano Science Center, Department of Chemistry, University of Copenhagen, Copenhagen Ø, 2100 Denmark; 6 Niels Bohr Institute, Blegdamsvej 17, University of Copenhagen, Copenhagen Ø, 2100 Denmark; 7 Feedstocks Division, Joint BioEnergy Institute, Emeryville, CA 94608 USA

## Abstract

Cytochrome P450 oxidoreductase (POR) is the primary electron donor in eukaryotic cytochrome P450 (CYP) containing systems. A wealth of ensemble biophysical studies of Cytochrome P450 oxidoreductase (POR) has reported a binary model of the conformational equilibrium directing its catalytic efficiency and biomolecular recognition. In this study, full length POR from the crop plant *Sorghum bicolor* was site-specifically labeled with Cy3 (donor) and Cy5 (acceptor) fluorophores and reconstituted in nanodiscs. Our single molecule fluorescence resonance energy transfer (smFRET) burst analyses of POR allowed the direct observation and quantification of at least three dominant conformational sub-populations, their distribution and occupancies. Moreover, the state occupancies were remodeled significantly by ionic strength and the nature of reconstitution environment, i.e. phospholipid bilayers (nanodiscs) composed of different lipid head group charges vs. detergent micelles. The existence of conformational heterogeneity in POR may mediate selective activation of multiple downstream electron acceptors and association in complexes in the ER membrane.

## Introduction

Cytochrome P450s (CYPs) are multifunctional heme-containing enzymes, which catalyze some of nature’s most complex and versatile chemical reactions^1,2^. In eukaryotes, they are anchored to the ER membrane and activated by their co-localized redox partner the NADPH-dependent cytochrome P450 oxidoreductase (POR). POR is a diflavin protein^3^ binding one molecule each of the FAD and FMN cofactors in distinct globular domains connected by a flexible hinge region^4^. Crystallographic structures have provided static snapshots of its modular architecture in a “compact”/closed conformation^4^ that facilitates inter-flavin (FAD →FMN) electron transfer and “open”/extended conformation, anticipated to mediate electron donation to downstream electron acceptors^5^. Several extended crystallographic structures have been characterized using deletion^6^ and cross-linked mutants^7^ of the N-terminal truncated POR and recently in its complex with heme oxygenase^8^. SAXS and NMR-based investigations^9^ in consensus with the crystal structure of the yeast-human chimeric POR^10^ have provided evidence of an alternate extended conformation. Conformational oscillation between compact and extended conformations plays a pivotal role in vectorial electron transfer of several redox-active biological systems^11^. Over the last decade, a wide array of ensemble biophysical studies have provided complementary evidences for concerted domain motions of POR^12^ and a highly dynamic equilibrium of its two conformers, which is regulated by redox potential^9,13^ and ionic strength^14,15^. Additionally, ELDOR studies of the disemiquinoid form of POR^16^ (FADH· FMNH·) and recent SAXS data^15^ have suggested the existence of a continuum of numerous conformations rather than discrete forms and envisioned a multidimensional energy landscape. The conformational plasticity of POR has been intricately associated with its catalytic function and biomolecular recognition towards a broad range of P450s^17^ some of which may be integrated with POR in dynamic metabolons^18–20^. Nevertheless, a comprehensive understanding pertaining to the number, abundance and relative distribution of POR conformational spectrum is yet to be attained.

Ensemble biophysical techniques obscure the heterogeneities of a complex system by delivering an average value. Direct observations of the dynamics of POR can be achieved by single molecule spectroscopy, which has emerged as a powerful tool to reveal heterogeneities in systems that remain elusive in bulk studies^21–24^. This enables quantification of the abundance of unsynchronized, transient conformational sub-states. Single molecule fluorescence resonance energy transfer (smFRET) is a technique, which acts as a ratiometric ruler to probe distance based molecular fluctuations such as conformational dynamics, protein folding or unfolding^25^. A smFRET-based study of the structurally related diflavin enzyme nitric oxide synthase (nNOSr) recently demonstrated that it adopts multiple open conformations^26^. As the conformational equilibria of the diflavin reductases vary substantially^27^ and kinetic analyses of electron flux in POR reveals different rate-determining steps in plant and mammalian enzymes^28^, it is intriguing to probe the structural diversity of POR.

The conformational dynamics of membrane-bound enzymes as a function of their reconstitution environment have also received much attention. Molecular dynamics simulation studies of rhodopsin^29^ and the integral outer membrane protein A bacterial (OmpA)^30^ protein illustrated increased fluctuation freedom in detergent micelles compared to phospholipid bilayers. However, selection of an appropriate model system for membrane proteins amenable for *in vitro* biophysical investigation remains a challenge. Classically, detergents have been utilized for solubilization of membrane proteins, though posing the caveat of being unilamellar and often disrupting protein structural and functional integrity^31^. Nanodiscs, discoidal patches of phospholipid bilayers encapsulated by an amphipathic scaffold protein (MSP), are monodisperse entities whose size and physico-chemical properties can be precisely manipulated^32,33^. It is noteworthy that the majority of existing information on POR conformational equilibrium is derived from either detergent solubilized or soluble versions lacking the N-terminal membrane anchor^34^. We have previously demonstrated that POR function and stability strongly depends on the presence of the membrane environment^35^. Neutron reflectivity data have also revealed a difference in the net conformational equilibrium value of full-length POR^36^ in ‘nanodiscs’ as compared to its truncated soluble form^9^.

In this report, a smFRET^37^ based approach was utilized to directly observe and quantify the conformational sampling of POR. A full length, double cysteine variant of *Sorghum bicolor* POR (N181C/A552C) solubilized in detergent micelles (DM) was site specifically labeled with the fluorophores Cy3 (donor) and Cy5 (acceptor) by maleimide chemistry. The dual labeled POR was reconstituted into nanodiscs, which contained phospholipids with 25% negative (ND1) and net neutral (ND2) head group charges. smFRET experiments of DM, ND1 and ND2 were carried out under freely diffusing conditions by confocal microscopy. In addition, the effect of ionic strength on the conformational sampling was investigated.

## Experimental

### Materials

All chemicals were of analytical grade and purchased from Sigma-Aldrich (Denmark) unless otherwise stated. 2′5′-ADP Sepharose for affinity purification, size exclusion chromatography (SEC) column Superdex 200 HR 10/30 for high-resolution preparative separation, Cy3 and Cy5 maleimide fluorophores and PD-10 desalting columns were purchased from GE Healthcare Life Sciences, Brøndby, Denmark. Phospholipids: 1,2-dilauroyl-*sn*-glycero-3-phosphocholine (DLPC) and 1,2-dilauroyl-*sn*-glycero-3-phosphorylglycerol sodium salt (DLPG) were purchased from Avanti Polar Lipids (Alabaster, USA). CriterionTGX Stain-Free (12%) precast gels and Bio-Beads SM-2 were obtained from Bio-Rad.

### Construct design

**Figure 1:**
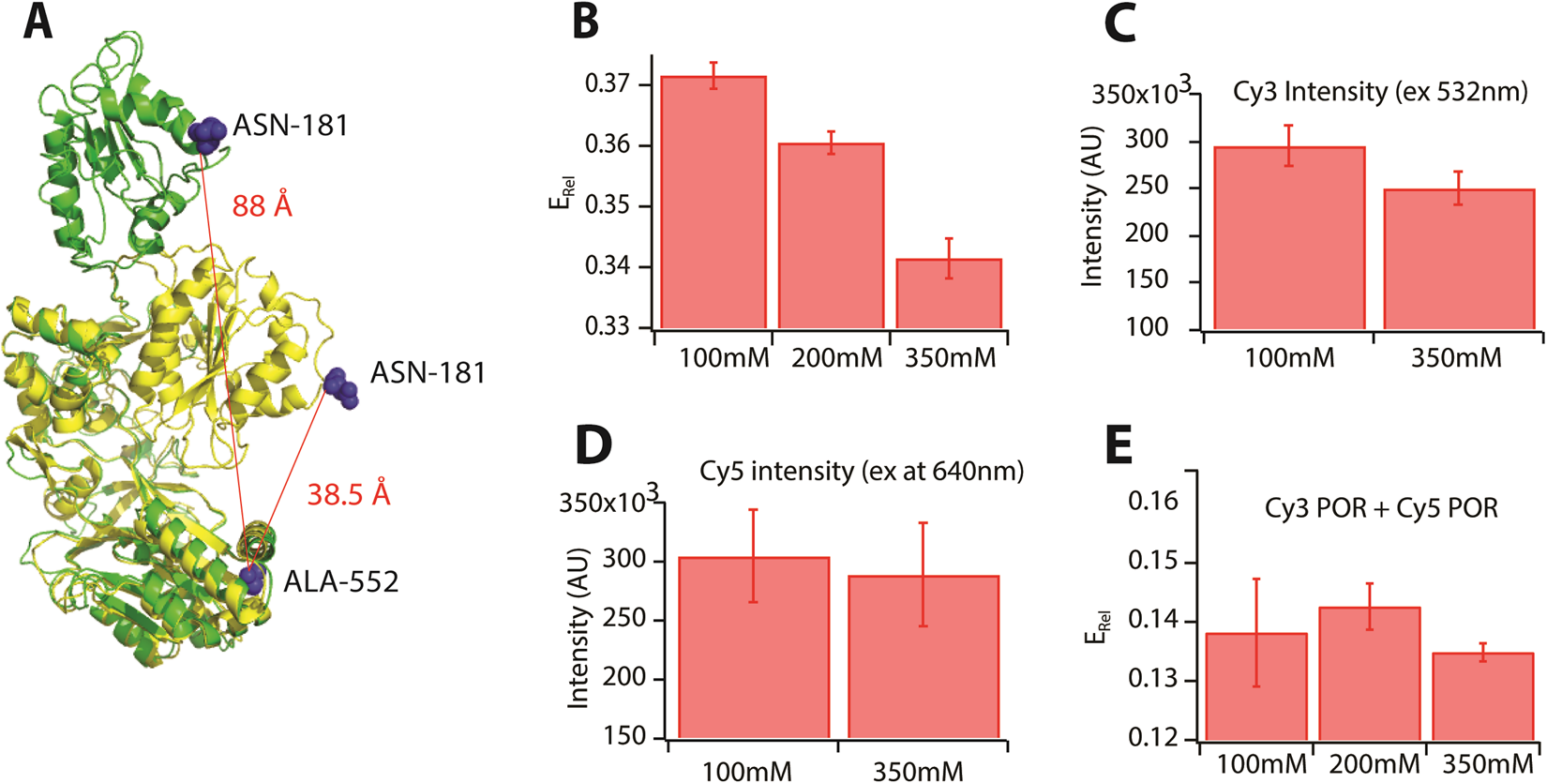
(**A**) An overlay of the compact and extended conformations POR based on homology models. The calculated Cα-Cα inter-residue distances between N181 and A552 are shown. (**B**) Average E_Rel_ dependence on ionic strength. Increasing ionic strength shifts the average conformational equilibrium to low FRET states. Control experiments showing minimal effect of salt concentration on the fluorescence properties of Cy3 (**C**), Cy5 (**D**) or the mixture Cy3 and Cy5 (**E**).

As the crystal structure of *Sorghum bicolor* POR is unavailable, homology modelling of *Sorghum bicolor* POR isoform 2b (GenBank: EES13592.1) was performed using the SWISS MODEL automated online server^38^. The compact conformation of *Sb*POR2b was based on POR from *Rattus norvegicus* as template (PDB ID: 1AMO_A^4^) while the open conformation was based on the yeast-human chimeric POR (PDB ID: 3FJO^10^), which displayed a sequence identity to *Sb*POR2b of 39% and 36%, respectively. Based on the 3D model structures, a pair of fully solvent accessible residues close to the FAD and FMN domains, but not directly involved in binding the flavin co-enzymes (N181 and A552 near the N- and C-termini, respectively) were selected for substitution with cysteine residues for fluorophore labeling. These residues would undergo major distance fluctuations during electron transfer with Cα-Cα distances of 38.5 Å to 88 Å (as calculated by the measurement wizard tool of PyMol software) in the compact and open conformations, respectively (Fig. 1A).

### Protein preparation and fluorophore labeling

The native surface exposed cysteine C536 was substituted to serine. The full-length, codon optimized POR gene containing specific cysteine mutations N181C/C536S/A552C was cloned into pET-52(b) expression vector (Genscript, USA). The full length POR mutant C536S/N181C/A552C was expressed in the *E. coli* BL21DE3 strain and purified using 2′5′-ADP Sepharose affinity chromatography as described previously^36^. The purified proteins were flash frozen in liquid nitrogen and stored at −80 °C until use. Fluorophore labeling of POR with an equimolar mixture of Cy3 and Cy5 mono-functional maleimides was carried according to manufacturer’s protocol (GE Healthcare). The free fluorophores were separated from the labeled proteins by gel filtration on PD-10 columns. Singly labeled POR with either Cy3 or Cy5 was prepared similarly for control experiments.

### Cytochrome-c activity assay

The dual labeled POR enzyme (0.045 µM) in buffer containing Tris 50 mM (pH 7.3) and 100 mM NaCl was incubated with 50 µM cytochrome *c* in a 1 mL cuvette for 2–3 min. After recording a baseline for 1 min, the reaction was initiated by addition of 1 mM NADPH and the amount of cytochrome *c* reduced was monitored by the time-dependent increase in absorbance at 550 nm on a Perkin-Elmer Lambda 650 UV/Vis spectrophotometer (ε_Cyt-c_ = 21.2 mM^−1^ cm^−1^). The slope of the curve in the linear region was used to determine the catalytic activity^39^.

### Ensemble FRET

Dual labeled POR in micelles was diluted to a concentration of 15 nM in standard buffer containing 50 mM Tris (pH 7.3), 100 mM NaCl and 0.1% Triton-X 100. A spectrofluorometer (Fluoromax, Horiba) was used to excite the sample at 532 nm (slit width = 5 nm) while the emission spectrum was recorded between 550–720 nm. Control experiments were carried out in parallel with single labeled Cy3 and Cy5 POR. For ionic strength experiments, POR was dissolved in buffer containing 50 mM Tris (pH 7.3) and 0.1% Triton-X 100 while the concentration of NaCl was varied from 100 mM to 350 mM. Fluorescence spectra were acquired as described above.

### Nanodisc reconstitution

POR reconstitution in nanodiscs was carried out following our recently published protocol^40^. In brief, the dual labeled POR solubilized in detergent was reconstituted into nanodiscs (NDs) containing the membrane scaffolding protein (MSP1E3D1) and either DLPC or a mixture of DLPC: DLPG (75/25 mol %) phospholipids. The molar ratios of MSP: Lipid: Protein was 1:120:0.1, which offers at least five-times molar excess of nanodiscs to POR enzyme. The incubation mixture also included 1 μM FMN and 1 μM FAD to replenish the enzyme with its co-factors. Detergent removal was facilitated by incubation with Bio-Beads for 4 h, which was followed by purification of nanodiscs by size exclusion chromatography (SEC) on a preparative HPLC (Shimadzu) equipped with a Superdex 200 Increase 10/300 GL (bed volume: 24 mL, flow rate: 0.5 mL/min). Cy3 POR and Cy5 POR were reconstituted into nanodiscs in a similar fashion.

### Surface preparation

Glass slides were cleaned thoroughly^35^ by sonication in milli-Q water, 70% ethanol and 2% hellmanex and stored in methanol until use. Prior to experimental use, the glass slides were dried under nitrogen flow, plasma etched by a Plasma cleaner PDC 32 G and placed in custom-made teflon chambers. The slides were repeatedly washed with 80 µL of the standard buffer [50 mM Tris (pH 7.3), 100 mM NaCl].

### Microscopy

FCS experiments on Cy3/Cy5 labeled POR were carried out in solution at nM concentrations. An inverted confocal microscope (Olympus IX71) was used along with an oil immersion objective HCX PL APO CS x 100 (NA 1.46) and two Avalanche Photodiode Detectors (APDs). Excitation at 543 nm was achieved by a diode laser (Thorlabs HGR020). The measurement was performed by focusing the laser 10 µm above the glass surface and the signals were collected in separate channels that were filtered with short-pass filters (Semrock) at above 650 nm for the donor channel and below 633 nm for the acceptor channel. The details of the microscopy setup used in this study and data acquisition are described in SI. (*Supplementary methods section M1*). All samples were measured at 100 mM and 400 mM NaCl concentrations and trajectories were collected for 300 sec.

### Data treatment

The detailed explanation of parameters used for thresholding, FCS data binning, autocorrelation data fitting, E_FRET_ calculation, and quantification of E_FRET_ distribution using Bayesian Information Criterion (BIC) are presented in SI (*Supplementary methods sections M2- M7*).

## Results

### Preparation of dual labeled POR for FRET

The full-length POR containing site-specific mutations N181C/C536S/A552C was labeled with Cy3 (donor) and Cy5 (acceptor) maleimides. Dual labeled POR in detergent micelles were confirmed by SDS-PAGE by multichannel Cy3 and Cy5 detection (Supplementary Fig. S1). The absorption spectrum of dual labeled POR revealed equimolar labeling of Cy3 and Cy5 (Supplementary Fig. S2). Furthermore, dual labeled POR was functional (Supplementary Fig. S3*)*, with a moderate loss (16%) in catalytic activity as compared to the unlabeled POR. This could be attributed to fluorophore labeling or loss of the loosely bound FMN cofactor during purification. Following, POR was reconstituted in nanodiscs and fractionated by size exclusion chromatography. Selected fractions were identified by absorbance at 280 nm (protein), 450 nm (flavins), 550 nm (Cy3) and 650 nm (Cy5) (Supplementary Fig. S4). The selected protein-loaded nanodisc fractions were combined and flash-frozen to be stored at −80 °C until use.

### Ionic strength regulates conformational equilibrium of POR

The steady state fluorescence spectral analyses of dual-labeled POR in detergent micelles revealed a relative FRET efficiency of 37% (Fig. 1B). The effect of ionic strength on the POR conformational equilibrium was studied by incrementing the amount of NaCl in the buffer from 100 mM to 350 mM. This led to a 8% decrease from 0.37 to 0.34 in relative FRET efficiency related to an extension of the conformation towards a more open structure (Fig. 1B), which is in agreement with earlier findings^41^. Control experiments confirmed that addition of salt did not alter the fluorescence properties of Cy3, Cy5 or the equimolar mixture of Cy3 and Cy5 labeled POR (Fig. 1C–E).

### Establishing a single molecule FRET platform to study conformational sampling

To directly observe and quantify the abundance of POR conformations we recorded smFRET by a parked beam confocal set-up. Experiments were performed using fluorescently labeled POR solubilized in detergent micelles (DM) or reconstituted in nanodiscs (NDs).

**Figure 2:**
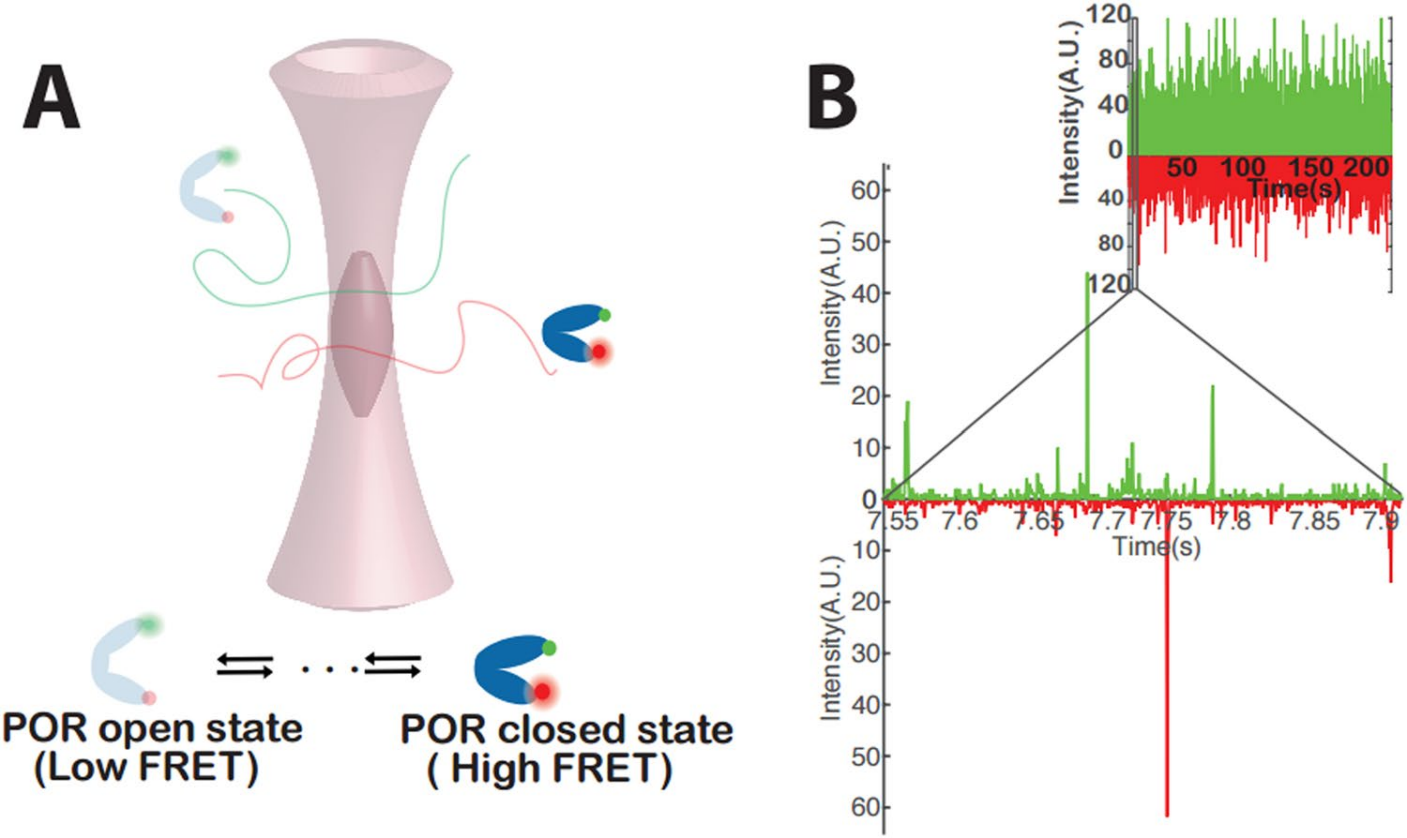
**(A)** A cartoon illustration of the experimental setup **(B)** Typical trajectory of detected photons recorded from ND reconstituted POR freely diffusing in solution. Coincident bursts correspond to an individual dual labeled POR traversing the diffraction-limited confocal volume. POR labeled with Cy3 or Cy5 only appear as single channel bursts. Inset shows an entire 250 s trace.

Our initial experiments were performed on POR in DM. Fluorescent bursts were collected from individual dual-labelled POR enzymes freely diffusing through the diffraction limited confocal volume (Fig. 2A). In typical trajectories, each coincident fluorescent burst in the Cy3 and Cy5 channels corresponds to an individual dual labeled POR enzyme diffusing through the confocal volume (Fig. 2B). We consequently evaluated the role of the reconstitution medium on the abundance and occupancy of POR conformational states. To do this, we recorded smFRET bursts of ND1 and ND2. To investigate the effect of ionic strength, a parallel set of smFRET experiments were conducted for DM, ND1 and ND2 in 400 mM NaCl.

### Direct observation and quantification of conformational states by smFRET

**Figure 3:**
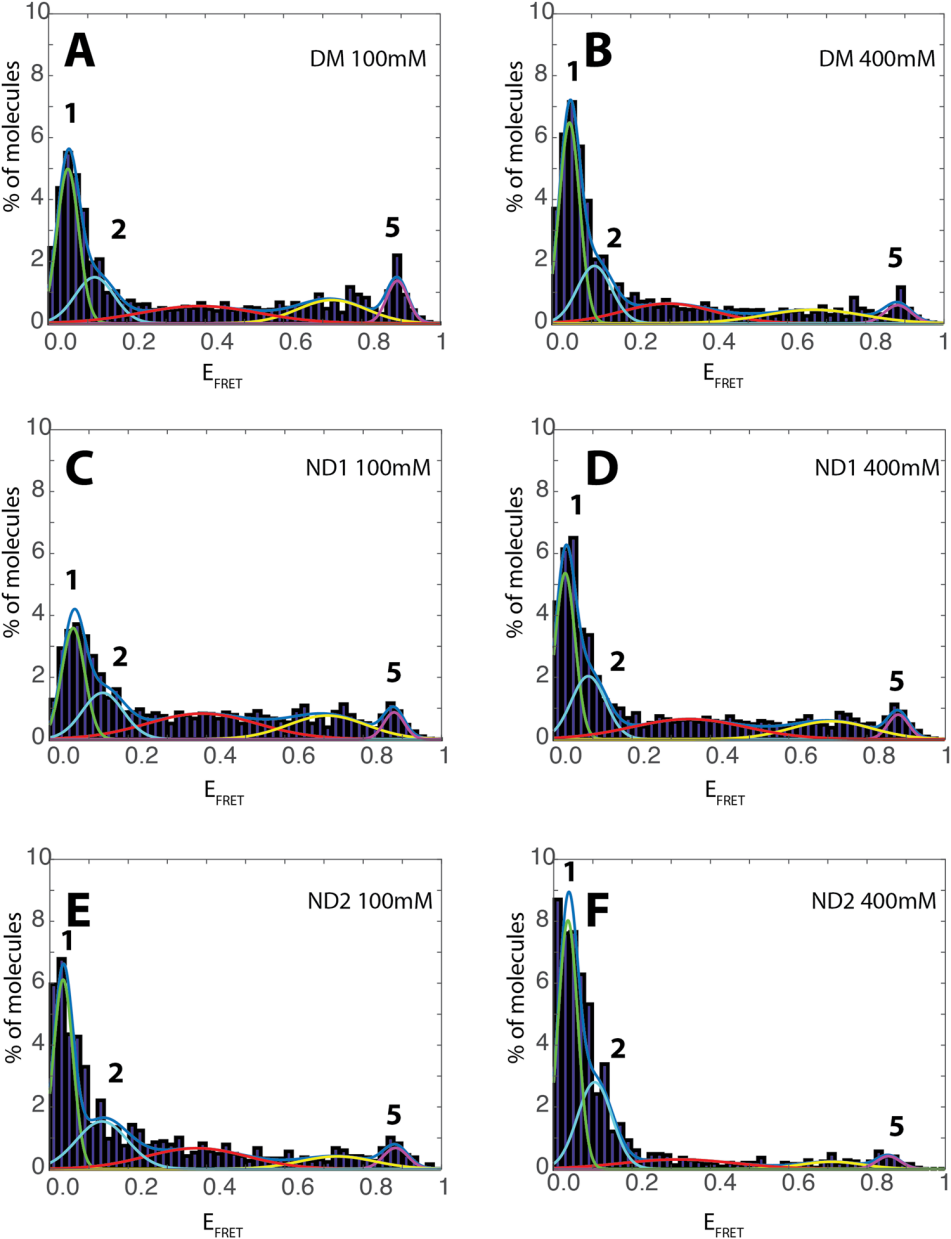
**(A**,**C** and **E)** E_FRET_ histograms of POR conformational sampling in detergent micelles (DM) and nanodiscs (ND1 and ND2) and (**B,D** and **F**) their dependence on increased ionic strength. The two “low FRET” peaks (1, 2) and high FRET peak (5) are indicated by line colors green, cyan and purple respectively.

Several fluorescence trajectories were collected and binned at 300 μs and 600 μs, for detergent and nanodiscs reconstituted POR respectively. The bin time resolution was in accordance with the residence time of POR derived by autocorrelation curves (Supplementary Fig. S5, Supplementary Table 1). Particles below the threshold^42^ were discarded from the analysis. The signals obtained after threshold application were utilized to determine the FRET efficiency (E_FRET_) after correcting for donor bleed through, detector efficiency and acceptor cross excitation (Supplementary Fig. S6, *method M1 and M6 for optimisation to avoid artefacts from dye photophysics*). The histogram of E_FRET_ for thousands of individual POR enzymes displayed a multimodal distribution indicating that POR adopts multiple conformations (Fig. 3A).

To correctly evaluate the number and the position of the states we; 1) acquired data analysing a high number of molecules (typically between 10^4^ and 7*10^4^), 2) fitted an increasing number of additive single Gaussian distributions to non-binned data^43,44^ (*Supplementary method M2*). A Bayesian information criterion^45^ (BIC) was employed to find the number of distributions that best described our data (Supplementary Fig. S7). This revealed at least three dominating non-zero Gaussian peaks at E_FRET_ 0.05 (peak 1), 0.12 (peak 2), 0.88 (peak 5) and two lower occupancy peaks (centered at ~0.4 and 0.6). The optimal fitting of data with five gaussians was also confirmed by Akaike information criterion (AIC) and the negative log likelihood in conjunction with Wilk’s theorem (Supplementary Table 2). Donor bleed through is also gaussian distributed and while accounted for by beta and gamma factor correction (Supplementary Fig. S6), some minor contribution might be observed in the low FRET states which might vary their ratios slightly, but not their existence (Supplementary Fig. S8). The E_FRET_ values for peaks 1, 2 and 5 were utilized to calculate their inter-dye distances (Supplementary Table 3*)*. The distances for peak 1 (86.6 Å) and peak 5 (38 Å) are in agreement with the Cα-Cα distances we expected from the homology models (i.e. 38.5 Å and 88 Å for the compact and extended conformations respectively, Fig. 1A). We note however that corrections for dye orientation may provide slightly different distances as compared to the homology model. Although we refrain ourselves from assigning defined conformational states based on single FRET derived distances, our single molecule data provide evidence for multiple conformations.

The FCS curves indicate the existence of low μs dynamics originating from POR and possibly from dye triplet state. Conformational dynamics occurring in time scales faster or comparable to the observation time^46,47^ may result in increased widths of the distributions as well as the observed plateau in intermediate E_FRET_ values. Quantification of these dynamics falls out of the scope of this paper. The additional intermediate conformations could thus originate from unresolved conformational dynamics occurring in time scales faster or comparable to the observation time^46,47^. Additionally, the existence of two extended (Fig. 3A, peaks 1 and 2) and a compact conformation (Fig. 3A, peak 5) suggests a landscape of multiple conformations. The surprisingly narrow width of the high FRET state may indicate a very stable compact conformation as compared to more dynamic extended conformations. This is in agreement with recent SAXS and NMR studies, which suggests that POR exists in a dynamic equilibrium between a rigid closed state and a highly flexible open statecharacterized by a large ensemble of conformations^15^. Indeed the distance dependent broadening of the low FRET states is beyond dye photophysics^48^ (Supplementary Fig. S9*)*. The two labeled POR domains (FAD and FMN) are connected via a flexible “hinge” domain. As this domain is highly flexible and partly disordered, this flexibility is expected to propagate to the POR domains introducing increased dynamics at greater distances of the extended conformations. These dynamics of the extended conformations would introduce the extra widening of the low E_FRET_ distributions while the high E_FRET_ distribution may corresponds to a well-defined compact conformation.

### Regulation of conformational sampling by ionic strength and membrane environment

**Figure 4:**
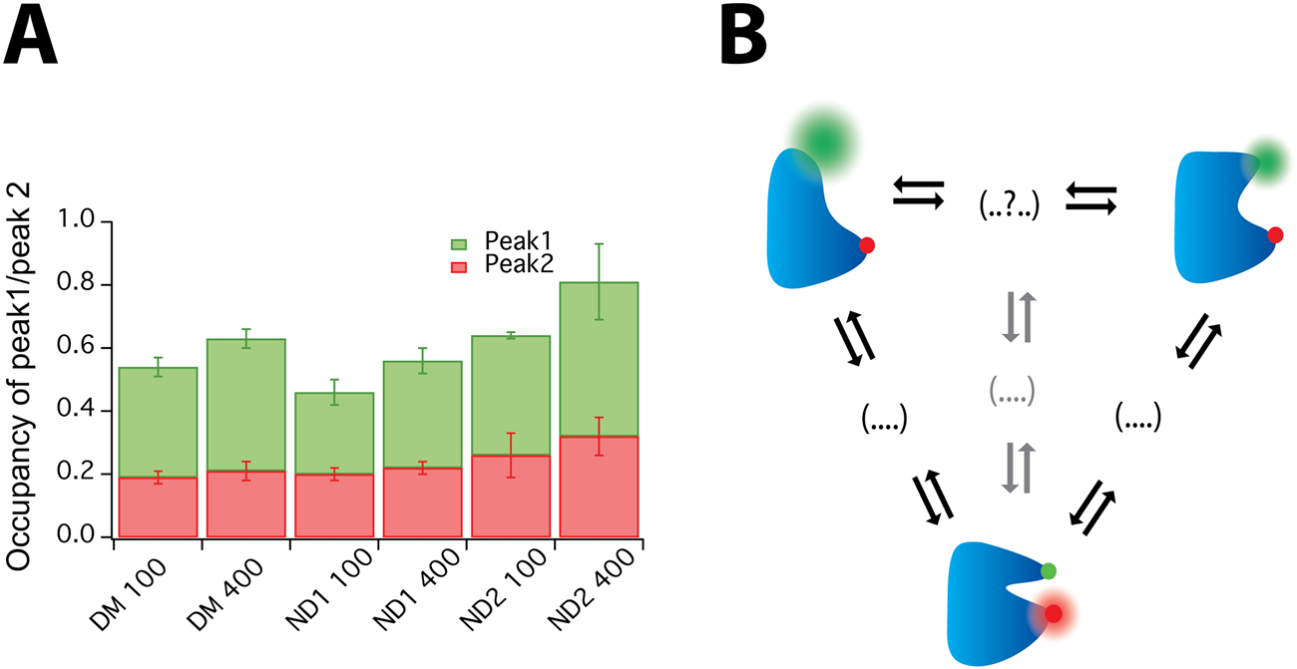
**(A)** Ratio of the occupancies of peaks 1 and 2 for POR in detergent micelles (DM) and nanodiscs (ND1 and ND2) as a function of ionic strength **(B)** An illustrative model of conformational sampling in POR.

POR conformational equilibrium is regulated by ionic strength and membrane environment as previously determined by ensemble studies. Therefore, we evaluated the role of the reconstitution medium on the abundance and occupancy of the herein described POR conformational states. To do this, smFRET bursts of ND1 and ND2 were recorded and the data were binned at 600 µs based on the residence time in the confocal volume (Supplementary Fig. S5C*)*. The positions and relative occupancies of the peaks did not change significantly upon moderate increasing in binning time (Supplementary Fig. 10A,B). In agreement with the data for POR in DM, BIC data analysis of ND1 and ND2 supported similar number of E_FRET_ states and peak positions, albeit with a remodelled conformational distribution (Fig. 3C,E, *supplementary methods* M7). Both the E_FRET_ and the state occupancy were found to strongly depend on the reconstitution medium. In order to rule out photo physical phenomenona of Cy3 when in close proximity to membranes or a protein^49^, we performed control experiments comparing Cy3 POR intensity distributions in detergent micelles and nanodiscs. Under our experimental conditions, we recorded a minute increase in the Cy3 intensity distribution in detergents as compared to nanodiscs (Supplementary Fig. S11). The relative occupancy of two extended conformations (sum of peaks 1 and 2) varied from POR reconstituted in DM (0.54 ± 0.03) to POR in NDs 0.47 ± 0.04 (13%) and 0.64 ± 0.12 (19%) for ND1 and ND2 respectively (Supplementary Table 4). More importantly, the relative ratio of occupancies for the two extended conformations was strongly dependent on the reconstitution medium and decreased from 1.8 for DM to 1.3 and 1.5 for ND1 and ND2 respectively (Fig. 4A) highlighting the strong effect of the reconstitution medium on membrane protein behaviour.

To investigate the effect of ionic strength, a parallel set of smFRET experiments were conducted in 400 mM NaCl. Control experiments revealed that addition of salt did not alter the residence time of POR in the confocal volume (Supplementary Fig. S5B,D) or the intensity of the donor or acceptor fluorophore (Supplementary Fig. S12). The Gaussian peak positions of the main peaks remained practically unchanged, however the occupancy of particular states varied significantly (Fig. 3B,D,F) indicating a regulatory mechanism enabling POR to redistribute the conformational landscape based on conformational selection^50^. For consistency, the data of ND2 were fit with 5 gaussians despite the practically complete shift towards the fully open states. The relative occupancy of two extended conformations (sum of peaks 1 and 2) increased 19% (from 0.54 ± 0.03 to 0.64 ± 0.02) for DM, 18% (from 0.47 ± 0.04 to 0.56 ± 0.03) for ND1 and 27% (from 0.64 ± 0.12 to 0.81 ± 0.16) for ND2 (Supplementary Table 4). This observed shift in equilibrium to extended states with increasing ionic strength is in agreement with previous reports proposing ionic strength as an efficient modulator of the POR conformational equilibrium and favouring open conformations^15^. Interestingly for all samples, this effect was mainly attributed to an increase in peak 1 corresponding to the most extended conformation of POR (Fig. 4A). This illustrates that the reconstitution medium affects the conformational landscape and its regulation by ionic strength.

## Discussion

In conclusion, our smFRET measurements of full-length POR in solution allowed the first direct observation and quantification of at least three dominant FRET states that in agreement with earlier studies support the existence of multiple conformational states. Our data does not distinguish between the “swinging” and “rotating” domain motion models^5^ or a delicate mixture of the two. The recently published 2.3 Å resolution crystal structure of *A. thaliana* POR (ATR2)^51^ in the compact conformation implies that the edge-to-edge distance between FAD and FMN is 10.6 Å, which is much longer than that in rat or yeast POR. Furthermore, a “half-closed” state with the FMN domain rotated away from the rest of the protein suggests that POR may sample a broader diversity of conformational structures. The existence of two “extended” conformers could facilitate alternative transition pathways to the compact conformation leading to altered rates of electron flux. We have previously described that POR fluctuates between two functional states by single turnover measurements^35^ involving enzymatic conversion of the prefluorescent substrate resazurin to resorufin^52^. These states may be associated with the two alternative transitions pathways and pinpoints a central feature of enzyme chemistry defining the relationship between structure and function. We speculate that the existence of two or more transition pathways (Fig. 4B) may mediate selective activation of >50 downstream P450 partners within dynamic metabolons, which stoichiometric outnumber POR by a factor 10–20^18,53^. Importantly, environmental challenges and local lipid and solvent environment may act as additional regulatory cues biasing conformational sampling pathways and consequently POR selectivity^18,19^.

An increase of the ionic strength caused POR to redistribute its conformational equilibrium and populate more open conformations (Fig. 4A). The proposition that the conformational ensemble of proteins varies between detergent micelles and bilayers has been validated for several systems^34,54,55^.

Our assay could be expanded to study the conformational repertoire of various missense mutants of POR in the functionally significant regions that lead to clinical symptoms in human beings^56^. Additionally, elucidating the conformational profiles of multiple POR isoforms found in plants^57^ would be of physiological relevance as compelling evidences reveal that class I PORs are constitutively expressed while class II includes those induced by stresses^58^. Integrating such heterogeneities may provide the basis for an improved understanding of P450-centric metabolic systems^59^, and the molecular mechanisms controlling encountered substrate channeling^18,60,61^.

## Electronic supplementary material

Supplementary Information
