# Supplementary material for: Direct observation of multiple conformational states in Cytochrome P450 oxidoreductase and their modulation by membrane environment and ionic strength
Source: Sci Rep. 2018 May 1;8:6817. doi: 10.1038/s41598-018-24922-x (PMC5931563; doi:10.1038/s41598-018-24922-x)
Supplement: Supplementary file 1 — Supplementary Information [file 41598_2018_24922_MOESM1_ESM.docx]

**Supplementary Information**

Direct observation of multiple conformational states in Cytochrome P450 oxidoreductase and their modulation by membrane environment and ionic strength

Krutika Bavishi^1,2,3,¶^, Darui Li^4,¶^, Stine Eiersholt^4^, Emma N. Hooley^5^, Troels C. Petersen^6^, Birger Lindberg Møller^1,2,3,*^, Nikos S. Hatzakis^3,4,*^ and Tomas Laursen^1,2,3,7,*^

^1^Plant Biochemistry Laboratory, Department of Plant and Environmental Sciences, University of Copenhagen, Thorvaldsensvej 40, Frederiksberg C, Copenhagen, Denmark-1871

^2^VILLUM Center for Plant Plasticity, Department of Plant and Environmental Sciences, University of Copenhagen, Thorvaldsensvej 40, Frederiksberg C, Copenhagen, Denmark-1871

^3^bioSYNergy, Center for Synthetic Biology, University of Copenhagen, Thorvaldsensvej 40, Frederiksberg C, Copenhagen, Denmark-1871

^4^Department of Chemistry & Nanoscience Center, University of Copenhagen, Thorvaldsensvej 40, Frederiksberg C, Copenhagen, Denmark-1871

^5^The Nano Spectroscopy Group, Nano Science Center, Department of Chemistry, University of Copenhagen, Universitetsparken 5, Copenhagen Ø, Denmark-2100

^6^Niels Bohr Institute, University of Copenhagen, Blegdamsvej 17, Copenhagen Ø, Denmark-2100

^7^Feedstocks Division, Joint BioEnergy Institute, Emeryville, CA 94608, USA

*¶The authors contributed equally to the work*

********Correspondence and request for materials should be addressed to blm@plen.ku.dk, hatzakis@nano.ku.dk or tola@plen.ku.dk*

**CONTENTS**

**I. SUPPLEMENTARY METHODS**

M1. Data acquisition

M2. Data handling and data treatment

M3. Thresholding setting

M4. FCS Data binning

M5. Autocorrelation function fitting

M6. Calculation of E_FRET_

M7. Fitting of gaussian distributions

**II. SUPPLEMENTARY FIGURES**

Fig.S1. Multichannel imaging of fluorescently labeled POR on TGX-stain free gel

Fig.S2. Absorption spectrum of dual labeled (Cy3 Cy5) POR

Fig.S3. Catalytic activity of dual labeled POR

Fig.S4. Purification of nanodiscs by size exclusion chromatography

Fig.S5. Autocorrelation curves of POR in DM and ND at 100mM and 400mM NaCl

Fig.S6. Calculation of correction factors (α, β, γ) for E_FRET_ determination

Fig.S7. BIC for determination of gaussian fittings

Fig.S8. Minimal contribution of D-only to recorded FRET signals

Fig.S9. Dependence of distribution width on distance of fluorophores

Fig.S10. Dependence of peak position and their relative occupancies on binning time

Fig.S11. Dependence of Cy3 fluorescence intensity variation on the reconstitution medium

Fig.S12. Effect of ionic strength on dye intensity

**III. SUPPLEMENTARY TABLES**

Table 1. FCS parameters

Table 2. Determination of optimal number of gaussians

Table 3. Inter-dye distances calculated for peaks 1, 2 and 5

Table 4. E_FRET_ peak positions and relative occupancies for POR in DM, ND1 and ND2

**IV.** **SUPPLEMENTARY REFERENCES**

**SUPPLEMENTARY METHODS**

**M1. Data acquisition**

Single molecule FCS detection of Cy3/Cy5 labeled POR were achieved through a home-made FCS set up using an inverted confocal microscope (Olympus IX71) with an oil immersion objective (HCX PL APO CS x 100 NA 1.46). Excitation was achieved at 543 nm through a cw solid-state diode laser (Thorlabs HGR020 543 nm) and filtered to ensure cleanness (Semrock FF01-543/3-25). Emission signals for the sample were separated by a dichroic at 552 nm (Semrock FF552-Di02-25x36), collected in individual channels, and filtered at 561 nm through two long pass filters (Semrock BLP01-561R-25). Subsequent short-pass and long-pass filtering were achieved at 650 nm (Semrock FF01-650/SP-25) and 633 nm (Semrock BLP01-633R-25) for the donor and acceptor channels respectively to avoid cross-talk. Photons from the two channels were detected separately through a pair of APDs (Picoquant, which were separated by a 50/50 dichroic mirror at 635 nm (Semrock Di02-R635-25x36). To avoid power dependent donor blinking behavior, we used the minimum possible power (4.01 µW at the objective, or 0.5 kW/cm2) enough to record reliable and reproducible signal.

**M2. Data handling and data treatment**

The data were fitted with a machine learning algorithm in Matlab (MATLAB R2015b). The gaussian fitting was done with a combination of k-clustering and expectation maximization (EM) algorithm^1^. The advantage of this algorithm is that the fittings are generated from probabilities of each data point belonging the individual gaussians, thus binning of data is not required. This is different from the typical non-linear least square fittings, where binning must first be used to generate a finite number of data points pre-fitting. We favored this method as the binning of data would introduce bias to shape of the distributions. To fit the data and determine the number of Gaussians we used a combination of k-clustering and expectation maximization (EM) algorithm. Details of the algorithm can be read here (^1^) but essentially this is an iterative process that calculates the likelihood of which Gaussians the data points belong to. This probability is used to assign the data points to the Gaussians, while initial positions of the Gaussians are first estimated by k-clustering.

**M3. Threshold setting**

The trajectories were subjected to a threshold using the well-established SUM^2^ (the sum of the intensities in the Cy3/Cy5 channels) method to eliminate the contribution of shot noise. The SUM threshold was chosen instead of AND and OR as it has been demonstrated to not bias the resulting distributions of E_FRET_^2^. The exact threshold values were empirically chosen based on the intensity profiles of the donor and acceptor dyes and were between T = 10 - 15 for the individual data sets. A home-made search algorithm was utilized to identify correlated signal in order to eliminate uncorrelated signal and artifacts caused by Cy3 bleed through and acceptor blinking, both of which would result in the appearance of a low FRET states in the data analysis.

**M4. FCS Data binning**

The raw FCS data were binned at appropriate time resolutions for the residence times of the proteins. The bin-width of the intensity trajectories were based on the diffusion time of the molecules as obtained from the autocorrelation functions (see Table 1). The bin times for the detergent and ND samples were selected to be 300 µs and 600 µs respectively to account for the error of the measurement. This was done to avoid the integration of multiple molecules into a single data bin, which would result in averaging of detectable dynamics. Previous research has also demonstrated the importance of this in the accuracy of conformational determination through FCS^3^.

**M5. Autocorrelation function fitting**

The autocorrelation function was fitted using the BIFL^®^ software whereby the following equation was used:


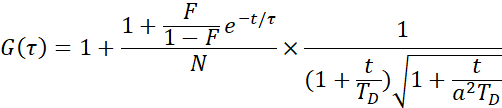


- *F* and *t* are triplet-state fraction and lifetime;
  – *N* is an average number of molecules in observation volume;
  – *T_D_* is the diffusion time;
- *α* is the ratio of axial to radial radii of the measurement volume

The autocorrelation plots in Fig S5 indicate the existence of low μs dynamics that may originate both from POR fast dynamics and possibly from dye triplet state, conformational dynamics occurring in time scales faster or comparable to the observation time^3, 4^. Extracting the proper timescales of protein dynamics requires corrections for cis-trans isomerization of both dyes, intersystem crossing, which extends beyond the scope of this study. Quantification of these dynamics falls out of the scope of this paper.

**M6. Calculation of E_FRET_**

To calculate E_FRET_, we corrected for the optics and detectors efficient γ factor and donor bleed through using the equation below.

To calculate the γ correction factor we performed a control experiment using a dsDNA labeled with Cy3/Cy5 with an inter-dye distance yielding E_FRET_ = 0.32. The sample (Custom oligos/Predefined DNA/RNA labeled with respectively Cy3 and Cy5) was purchased from IBA Life Sciences. By fitting a Gaussian distribution to the distribution of γ values (see figure S6A) we found γ = 2.56.

To quantify correction β factor, we quantified the contribution of donor bleed-through to the resulting E_FRET_ distribution (see figure S6B) under otherwise identical imaging conditions.

We found no measurable contribution of direct acceptor excitation by the 543 nm laser under the power regime that we have adopted (see fig S6C) so used α =0

The E_FRET_ correction accounts for intensity contribution arising from inactive acceptor providing donor only signal^5^. Moreover, if acceptor blinking or acceptor sampling the “dim” state^6^ was the underlying reason for the two neighboring distributions of the low E_FRET,_ one would record two neighboring distributions for the high E_FRET_ state as well. The experimental data however clearly show a well-defined distribution at high E_FRET_ confirming that the possible acceptor photophysics in our experimental condition is minimal. It is also noteworthy that the cis-trans isomerization of Cy3 is primarily taking place in the low microsecond time scales^7^, both intersystem crossing as well as the isomerisation decay are within <30µs (4 and 20µs respectively) while average orientiation factor (kappa) can cause to an error of up to ~10%^8^. Cy5 isomerisation and photophysics occur across similar time scales^6^. In both cases these are laser power dependent and are practically minimal in our experimental conditions (*Supplementary methods M1*).

**M7. Fitting of Gaussian distributions**

Gaussian distributions were used to fit the histograms to identify individual populations of E_FRET_ states. The histograms were fitted using the Expectation Maximization (EM) algorithm along with k-means clustering to determine the initial guesses. These two machine learning algorithms are often used in data analysis where the number of identifiable variables in the data sets are unknown and were chosen to avoid any assumptions would systematically limit the scope of the fits.

In order to avoid over-fitting, the Bayesian Information Criterion (BIC) was utilized. BIC is calculated by [BIC = -2 • lnL + *k* • ln(n)], where L is the maximized likelihood function based on the number of free parameters and the observed data set, *k* is the number of parameters to be analyzed and n is the number of data points.

To further compare the validity of this method we also employed the well-known model comparing algorithms of Akaike Information Criterion (AIC)^9^ and Wilk’s theorem in conjunction with the negative loglikelihoods^10^. Although these two methods are not as strong as BIC in terms of punishing overfitting, they nonetheless show that 5 gaussians are the optimal number of fits. (Supplementary Table 2)

The lower-bound for the widths of the fitted distributions (σ) was determined by fitting the E_FRET_ distribution obtained of Cy3 labeled POR. The width of these distributions includes shot noise contributions as well as well as putative cis-trans dye motions. Each fit was replicated ten times to ensure its accuracy. Gaussian fitting with the aforementioned algorithms on this data allowed for the determination of the minimum σ of conformational distributions that can be identified by the analysis. The minimum width was chosen to be σ = 0.0006. On the basis of this the only restriction on Matlab algorithm was the minimum width. The low number of data points for high FRET values of ND2 provides poor fitting so the same fitting conditions as for ND1 and DM were applied for comparison. The software calculated the number of Gaussians as well as their widths.

**SUPPLEMENTARY FIGURES**


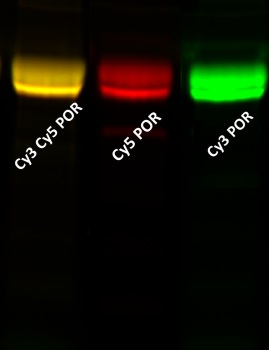


**1 2 3**

**Fig. S1.** Multichannel imaging in TGX stain free gel for Cy3 Cy5 POR (dual labeled, lane 1), Cy5 POR (acceptor only, lane 2) and Cy3 POR (donor only, lane 3)

**
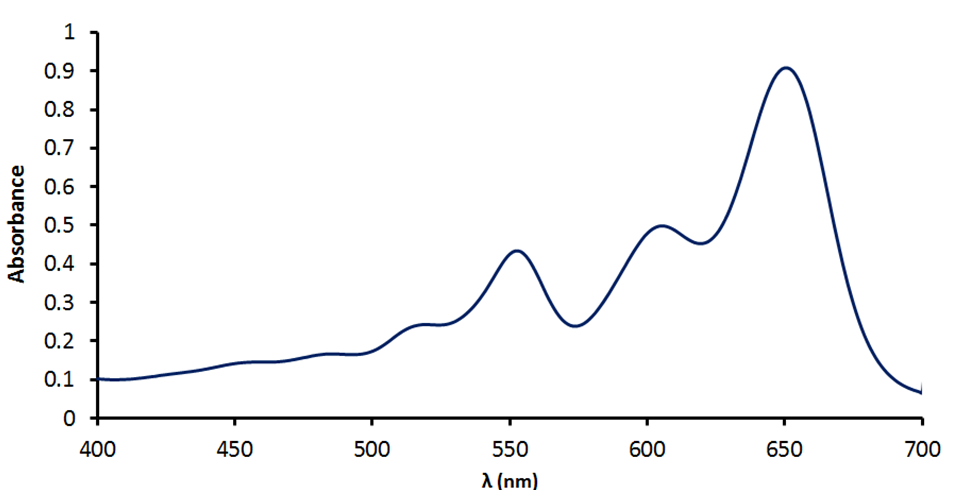
**

**Fig. S2.** The absorption spectrum of dual labeled POR revealed maxima at 452 nm (POR flavins), 552 nm (Cy3) and 650 nm (Cy5) indicating 1: 0.4: 0.5 stoichiometric ratios of POR:Cy3:Cy5 (ε_POR_, ε_Cy3_, ε_Cy5_ are 21.2, 150 and 250 mM^-1^ cm^-1^ respectively).

**
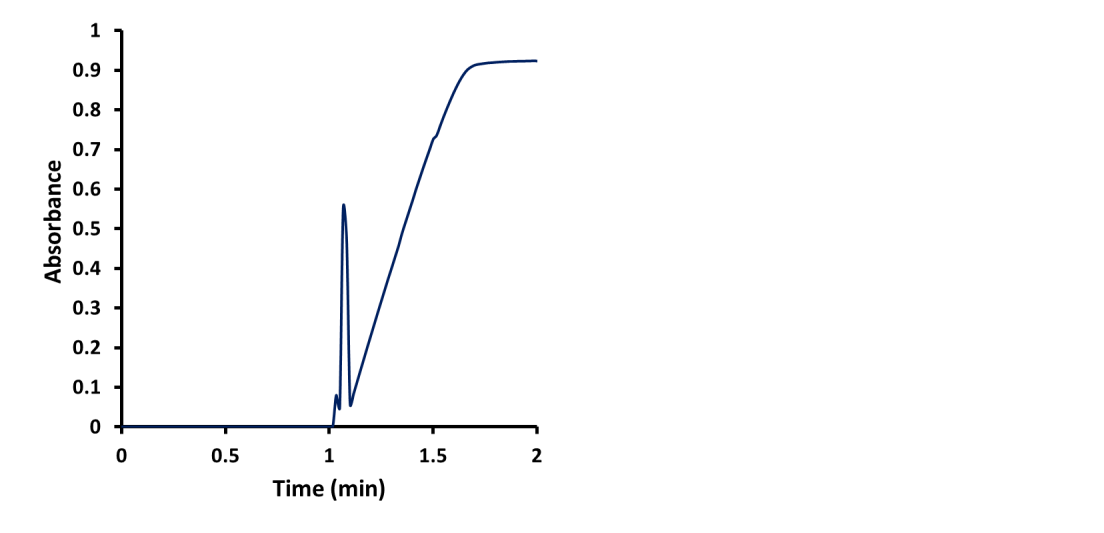
**

**Fig. S3**. The catalytic activity of Cy3 Cy5 dual labeled POR was measured by reduction of cytochrome *c*. After recording a baseline for 1 min, the reaction was initiated by addition of 1 mM NADPH (addition seen as a spike in the curve) and the amount of cytochrome *c* reduced was monitored by the time-dependent increase in absorbance at 550 nm The slope of the curve in the linear region was used to determine the catalytic activity of the dually labeled POR (k_cat_ =1,777 min^-1^). The activity of POR prior to labeling was k_cat_ =2,105 min^-1^ (not shown).


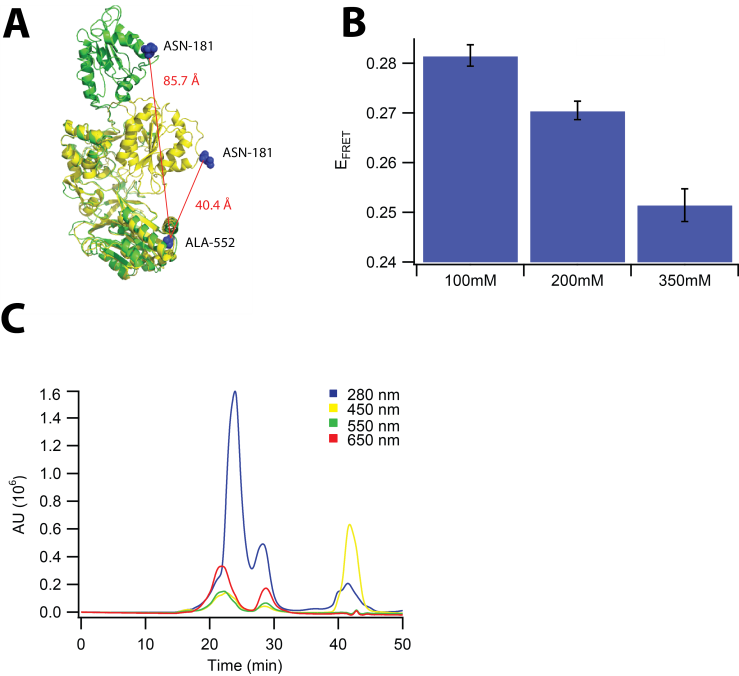


**Fig. S4.** Purification of nanodiscs by size exclusion chromatography. Relevant fractions are identified by recording absorbance at 280 nm (protein), 450 nm (flavins), 550 nm (Cy3) and 650 nm (Cy5).


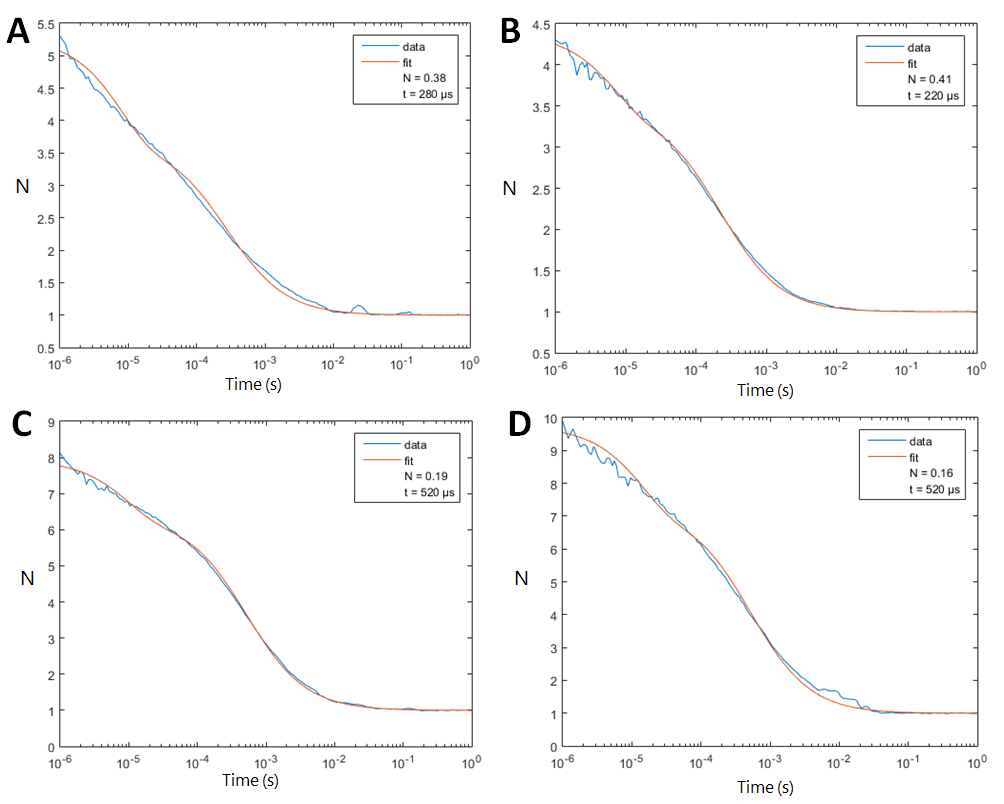


**Fig. S5**. Autocorrelation curves of POR in DM and ND1 at 100 mM NaCl **(A, C)** and 400 mM NaCl **(B, D)**. Data are fit for a single diffusing component that may blink.


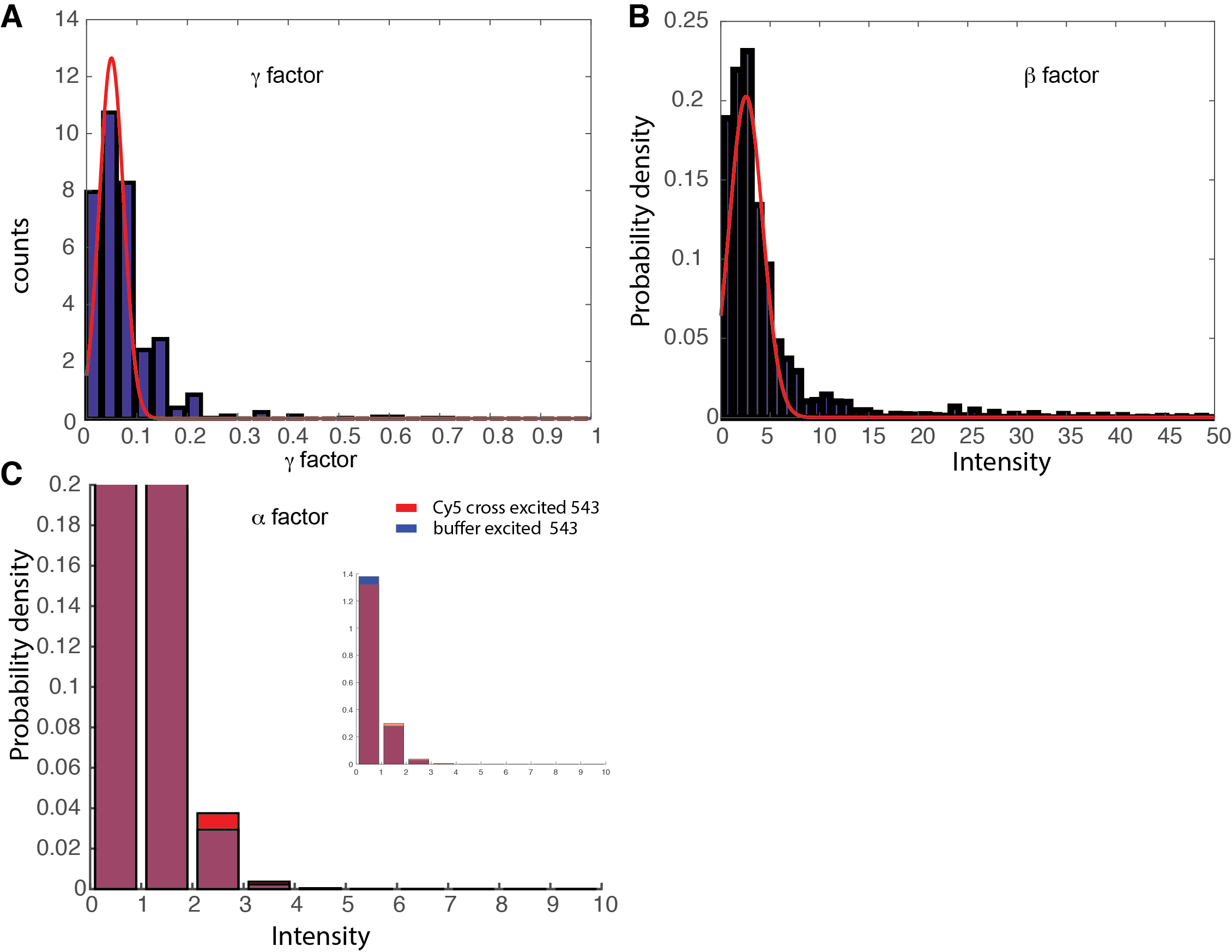


**Fig. S6. Corrections for E_FRET_ calculation (A)** γ factor calculation by dsDNA labeled with Cy3/Cy5 with an inter-dye distance yielding E_FRET_ = 0.32. (**B)** Donor bleed through was quantified by imaging donor only POR in identical imaging conditions (**C)** We found no measurable contribution of direct acceptor excitation by the 543 nm laser under the power regime that we have adopted so used α =0. Comparison to a sample of buffer only is shown.


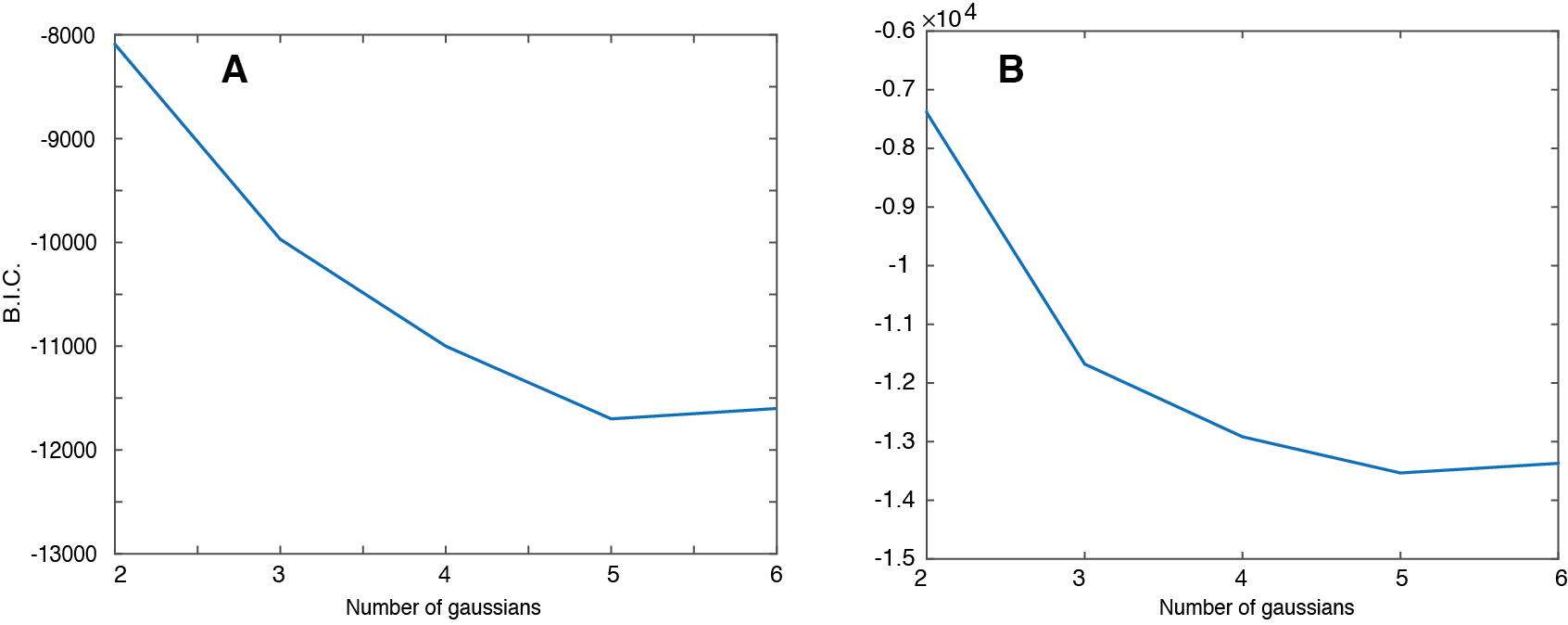


**Fig. S7.** Representative graph showing how the Bayesian Information Criterion (BIC) is used to determine the optimal number of Gaussian fittings to the histograms. **(A)** Data from DM 100 mM NaCl sample. **(B)** Data from ND1 100 mM NaCl sample. In all cases the decrease in BIC from 4 to 5 Gaussians is larger than 100 and up to 700, convincingly illustrating that 5 Gaussians optimally fit the distributions^11^.


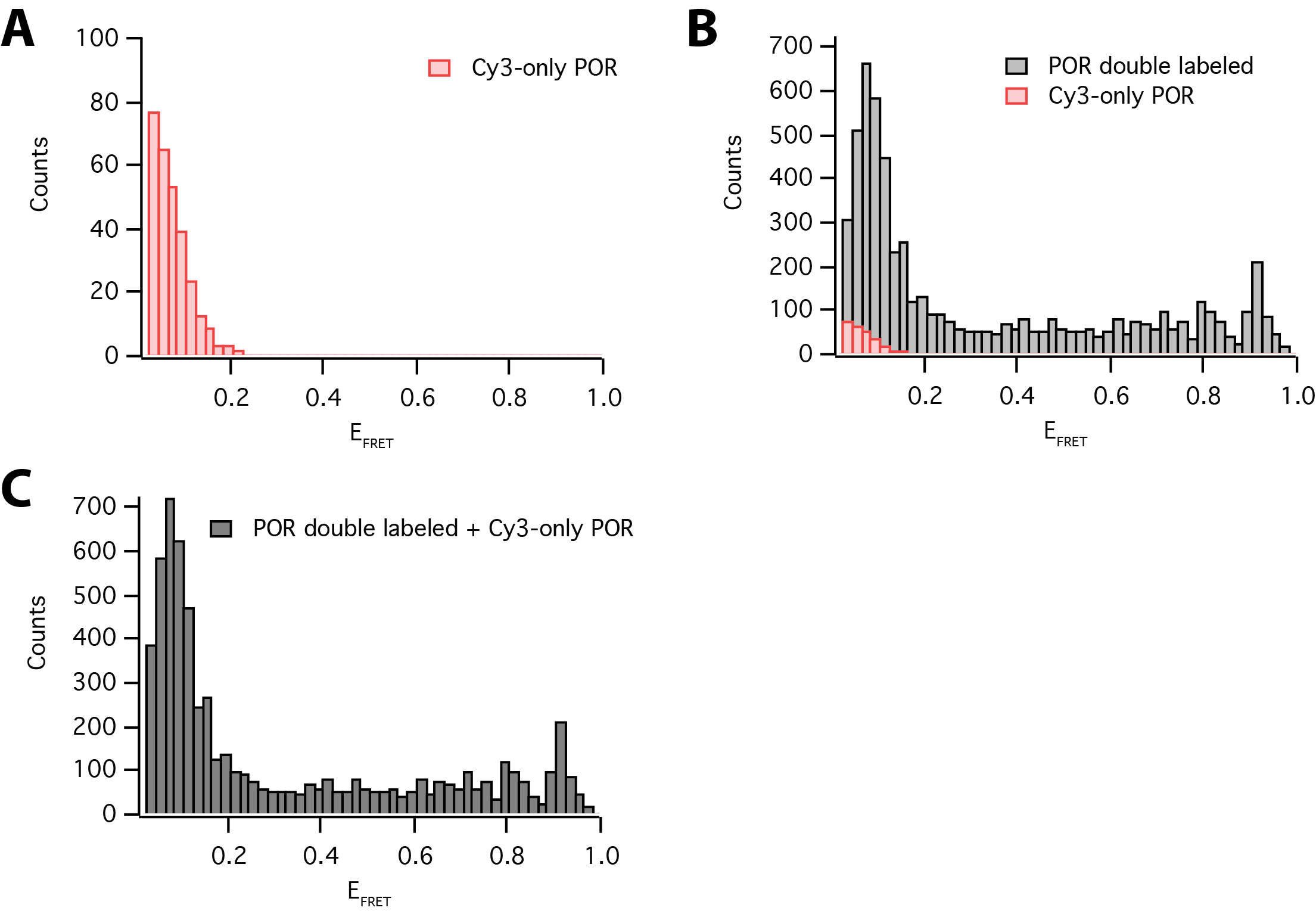


**Fig. S8.** Minimal contribution of D-only to recorded FRET signals. A. Control measurements of Cy3-only labeled POR treated in otherwise identical conditions and corrected for beta and gamma show a distribution centered around zero values as expected. The concentrtion of D-only used was ~25% of the double labeled, corresponding to the amount of D-only labeled POR under our conditions. B. Overlay of Cy3-only signal with POR FRET display indeed the discrete D-only peak as compared to our FRET distribution. C. Mixing of D-only and doubly labeled POR data with a ratio of ~1:4, which would correspond to the amount of D-only in the labeled sample, indeed reveals that the minimal contribution of D-only signal might slightly vary the ratios of low FRET states but not their existence.


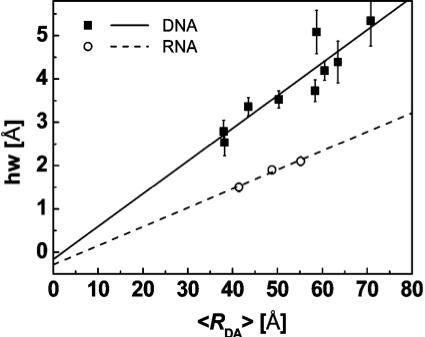

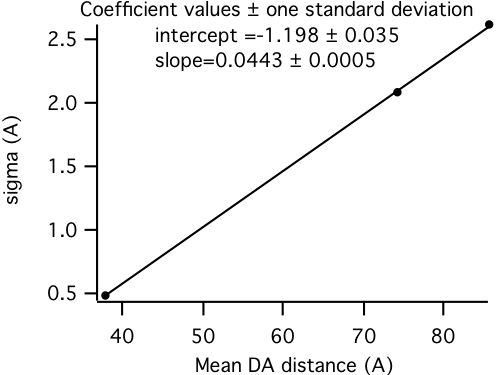


**Fig. S9. (A)** Mean donor-acceptor distances and the apparent distance distribution half widths ( σ) measured for a series of POR in DM at 100 mM NaCl showing a linear increase in the width of distributions with increasing distance of fluorophores in agreement with earlier reports^12^. The fact that the linear fit of our data does not cross the origin indicating that we record some distance dependent broadening beyond dye photophysics^12^ **(B)** Mean donor-acceptor distances and the apparent distance distribution half widths (hw) measured for a series of labeled dsDNA and dsRNA fragments. Reprinted (adapted) with permission from Kalinin et al^12^. Copyright (2010) American Chemical Society.

**
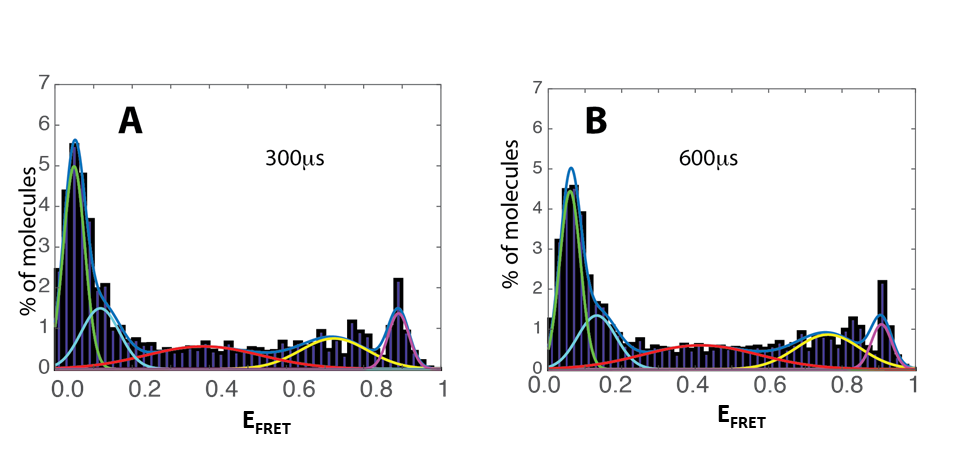
**

**Fig. S10.** The peak positions of the E_FRET_ distribution and relative occupancies of POR in DM at 100 mM NaCl are independent of the binning time used as seen in **(A)** at 300 µs and **(B)** at 600 µs


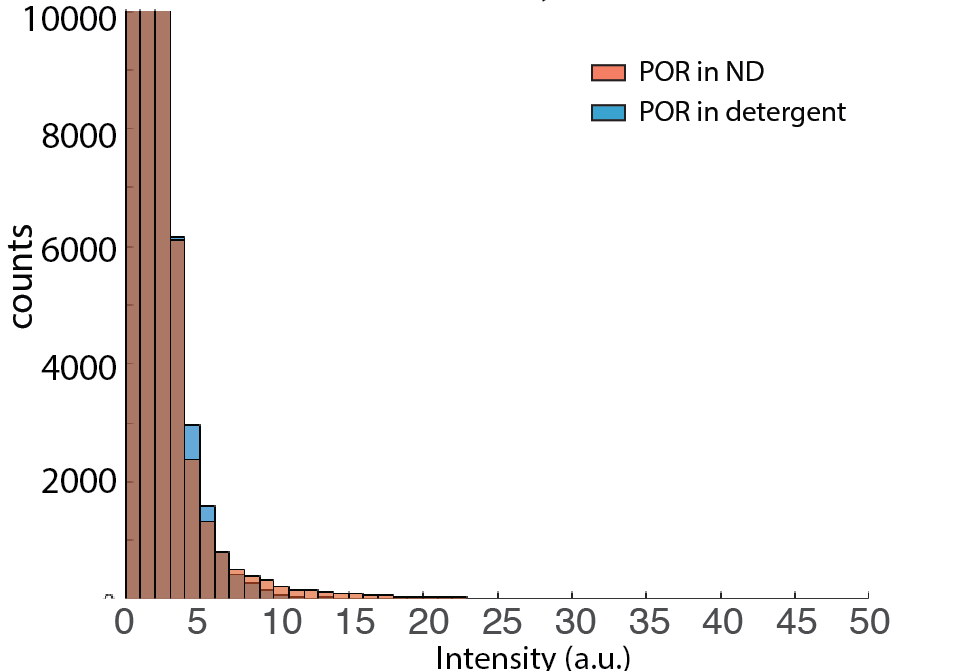


**Fig. S11.** Environmental dependence of POR conformational sampling is not due to Cy3 fluorescence intensity variation by the reconstitution medium. Intensity of Cy3 remains practically unchanged for POR in detergent or reconstituted in nanodiscs.


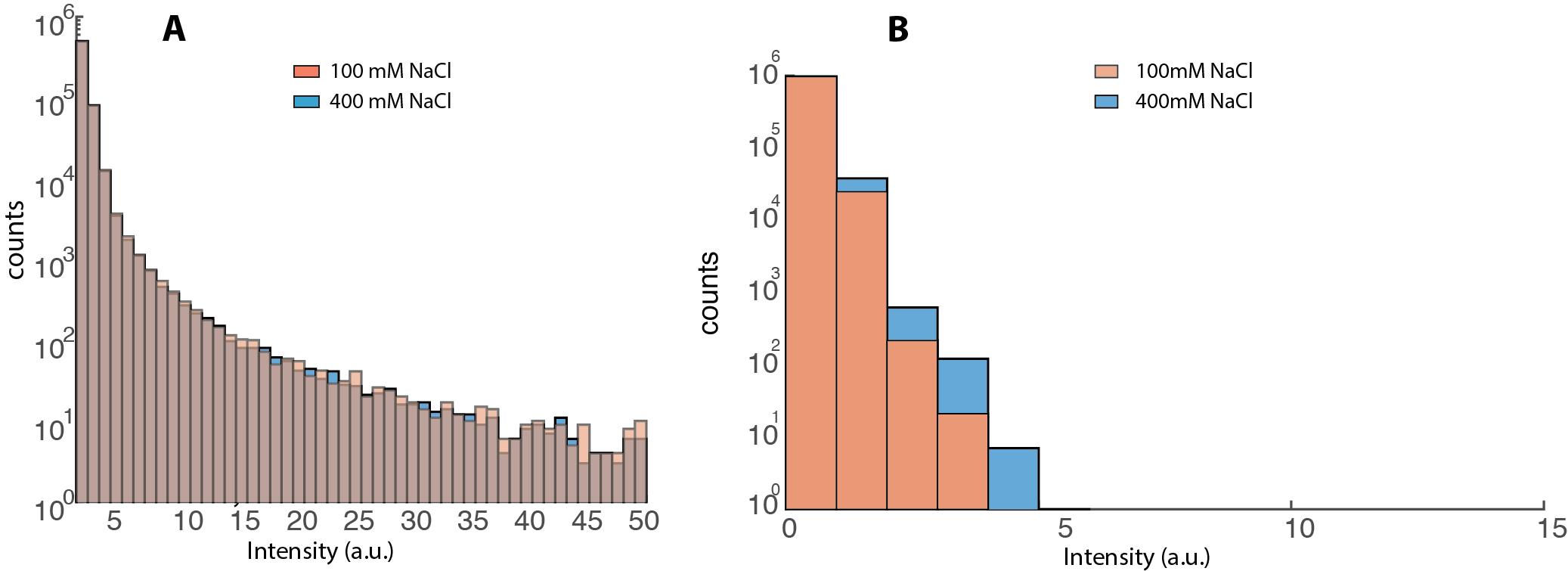


**Fig. S12.** Ionic strength does not alter dye photophysics. Intensity of **(A)** Cy3 POR and **(B)** Cy5 POR when excited with 543 nm laser remains unchanged when ionic strength is increased from 100 mM to 400 mM NaCl.

**SUPPLEMENTARY TABLES**

| **Condition** | **N** | ***T_Dj_*** |
| --- | --- | --- |
| DM (100mM) | 0.38 | 2.8 × 10^-4^ |
| DM (400mM) | 0.41 | 2.2 ×10^-4^ |
| ND1 (100mM) | 0.19 | 5.2 × 10^-4^ |
| ND1 (400mM) | 0.16 | 5.2 × 10^-4^ |
| ND2 (100mM) | 0.26 | 2.7 × 10^-4^ |
| ND2 (400mM) | 0.21 | 2.8 × 10^-4^ |

**Supplementary Table 1:** Autocorrelation parameters from FCS analysis.

| **Number of gaussians (N)** | AIC | BIC | -2ln | -2ln_N-1_ / -2ln_N_ |
| --- | --- | --- | --- | --- |
| 3 | -10000 | -9970 | -5020 | 950 |
| 4 | -11100 | -11000 | -5,550 | 530 |
| 5 | -12000 | -11700 | -5930 | 380 |

**Supplementary Table 2.** Three different methods were used to determine the optimal number of Gaussians. The AIC, BIC, and the negative log likelihood in conjunction with Wilk’s theorem. All three methods agree that 5 is the best fit. Data are shown for DM 100 mM NaCl. The fitting with 6 Gaussians is not presented as a limit for minimum Gaussian width (sigma) was used in the fitting algorithm, which causes the fitting of 6 Gaussians to not converge properly.

| **Condition** | Distances for peaks 1,2 & 5 (Å) |
| --- | --- |
| DM (100 mM) | 86.6 ± 1.6, 73.9 ± 1.5, 38.0 ± 0.4 |
| DM (400 mM) | 90.0 ± 2.0, 75.1 ± 2.7, 40.2 ± 1.6 |
| ND1 (100 mM) | 83.8 ± 1.4, 71.7 ± 1.6, 38.0 ± 0.8 |
| ND1 (400 mM) | 94.6 ± 2.3, 77.9 ± 1.4, 38.0 ± 0.8 |
| ND2 (100 mM) | 94.6 ± 3.2, 72.8 ± 1.2, 40.7 ± 1.1 |
| ND2 (400 mM) | 90.0 ± 3.2, 75.1 ± 1.8, 42.5 ± 2.9 |

.

**Supplementary Table 3:** Inter-dye distances calculated for peaks 1, 2 and 5

| **Condition** | **Peak center** | **Relative occupancy** |
| --- | --- | --- |
| DM  100 mM | 0.05 ± 0.01, 0.12 ± 0.01, 0.39 ± 0.02, 0.73 ± 0.00, 0.88 ± 0.01 | 0.35 ± 0.03, 0.19 ± 0.02, 0.21 ± 0.03, 0.14 ± 0.03, 0.11 ± 0.02 |
| DM  400 mM | 0.04 ± 0.01, 0.11 ± 0.02, 0.31 ± 0.07, 0.63 ± 0.09, 0.84 ± 0.03 | 0.42 ± 0.03, 0.21 ± 0.03, 0.20 ± 0.01, 0.08 ± 0.01, 0.09 ± 0.01 |
| ND1  100 mM | 0.06 ± 0.01, 0.14 ± 0.01, 0.39 ± 0.03, 0.71 ± 0.03, 0.88 ± 0.02 | 0.26 ± 0.04, 0.20 ± 0.02, 0.29 ± 0.05, 0.19 ± 0.02, 0.06 ± 0.02 |
| ND1  400 mM | 0.03 ± 0.01, 0.09 ± 0.01, 0.35 ± 0.03, 0.71 ± 0.03, 0.88 ± 0.02 | 0.34 ± 0.04, 0.22 ± 0.02, 0.24 ± 0.02, 0.15 ± 0.04, 0.05± 0.01 |
| ND2  100 mM | 0.03 ± 0.01, 0.13 ± 0.01, 0.38 ± 0.04, 0.73 ± 0.13, 0.88 ± 0.03 | 0.38 ± 0.08, 0.26 ± 0.04, 0.21 ± 0.04, 0.1 ± 0.04, 0.05 ± 0.04 |
| ND2  400 mM | 0.04 ± 0.01, 0.11 ± 0.01, 0.31 ± 0.02, 0.71 ± 0.08, 0.85 ± 0.08 | 0.49 ± 0. 10, 0.32 ± 0.06, 0.11 ± 0.05, 0.05 ± 0.01, 0.03 ± 0.01 |

**Supplementary Table 4.** The E_FRET_ peak positions and relative occupancies of POR in detergent micelles (DM), anionic nanodiscs (ND1) and net neutral nanodiscs (ND2) and their regulation by ionic strength.

**SUPPLEMENTARY REFERENCES**

1. C. B. Do and S. Batzoglou, *Nat. Biotechnol.*, 2008, **26**, 897.

2. A. A. Deniz, T. A. Laurence, M. Dahan, D. S. Chemla, P. G. Schultz and S. Weiss, *Annu. Rev. Phys. Chem.*, 2001, **52**, 233-253.

3. I. V. Gopich and A. Szabo, in *Single-Molecule Biophysics*, John Wiley & Sons, Inc., 2011, DOI: 10.1002/9781118131374.ch10, pp. 245-297.

4. B. Schuler and W. A. Eaton, *Curr. Opin. Struct. Biol.*, 2008, **18**, 16-26.

5. T. Ha and P. Tinnefeld, *Annu. Rev. Phys. Chem.*, 2012, **63**, 595-617.

6. Z. Huang, D. Ji, S. Wang, A. Xia, F. Koberling, M. Patting and R. Erdmann, *J. Phys. Chem. A*, 2006, **110**, 45-50.

7. K. Jia, Y. Wan, A. Xia, S. Li, F. Gong and G. Yang, *J. Phys. Chem. A*, 2007, **111**, 1593-1597.

8. D. B. VanBeek, M. C. Zwier, J. M. Shorb and B. P. Krueger, *Biophys. J.*, 2007, **92**, 4168-4178.

9. H. Akaike, *IEEE Trans. Autom. Control*, 1974, **19**, 716-723.

10. S. S. Wilks, *Ann. Math. Statist.*, 1938, **9**, 60-62.

11. R. E. Kass and A. E. Raftery, *J. Am. Stat. Assoc.*, 1995, **90**, 773-795.

12. S. Kalinin, E. Sisamakis, S. W. Magennis, S. Felekyan and C. A. M. Seidel, *J. Phys. Chem. B*, 2010, **114**, 6197-6206.
